# Supplementary material for: Are carbon emissions trading and green financial instruments synergistic? -Comprehensive quantitative research based on content analysis
Source: PLoS One. 2024 Mar 7;19(3):e0298601. doi: 10.1371/journal.pone.0298601 (PMC10919601; doi:10.1371/journal.pone.0298601)
Supplement: S1 Data — (ZIP) [file pone.0298601.s001.zip › Data/地级市Z_F工作报告.zip!/╔╜╬≈/╜·│╟╩╨/└┤╫╘╣1⁄2╓┌║┼í╛┬φ┐╦╩2╛▌═°í┐_2004.docx]

各位代表：

　　现在，我代表市人民政府向大会作政府工作报告，请予审议，并请市政协委员和其他列席人员提出意见。

　　一、2003年政府工作回顾

　　2003年是我市全面建设小康社会进程中不平凡的一年。面对突如其来的非典疫情和频繁发生的自然灾害,在中共晋城市委的正确领导下，全市各级政府和广大人民群众以邓小平理论和“三个代表”重要思想为指导，认真贯彻中央和省委省政府的各项要求，紧紧围绕“一争两转五突破”的工作部署和“11445”的发展战略，万众一心，奋力拼搏，坚持一手抓抗击非典不松劲，一手抓经济建设不动摇，取得了经济建设和各项社会事业持续快速健康发展的新成就。

　　1、主要经济指标全面超额完成。全市生产总值完成198.5亿元,比上年增长13.5%；财政总收入突破30亿大关,达到30.1亿元, 增长35.2%。比2000年翻了一番多,六个县（市、区）均突破亿元。一般预算收入10.3亿元，增长31.3%；城镇居民人均可支配收入7071元，增长15.8%;农民人均纯收入2942元，增长8.7%；城镇登记失业率1.7%，低于省控指标2.3个百分点；人口自然增长率2.67‰,远低于7.1‰的年度目标；市区空气质量二级以上天数达到172天，增加113天。市四届四次人代会确定的各项目标圆满完成。

　　2、经济结构调整进展良好。粮食总产量达72.4万吨。蔬菜大棚发展到1510栋,总产量达到30.8万吨。畜牧业作为现代农业的带动产业开始启动。生猪、牛、羊、蚕茧分别达到40万头、7万头、57万只和2927吨。陵川中药材基地发展到8万亩。绿色农业和无公害农业示范基地分别达16万亩、25.6万亩。13个农业产业化重点项目建成投产。植树造林25.9万亩。区域特色农业初显端倪。“工业立市”理念普遍确立,“四大基地”建设进展顺利。煤炭行业坚持“关小改中建大”,一批大中型矿井相继投产,煤炭加工转化率达到25%。煤转肥、煤转电战略快速推进。两个“1830”项目已按期完成,基本形成103万吨尿素生产能力。天脊“4060”、晋丰“3652”和兰花“1830”三个化肥项目顺利进行。阳电二期2×60万千瓦和兰花阳城、高都两个2×13.5万千瓦项目开工建设,热电联供、鲁能电厂正在组织实施。冶铸业在关小的同时,建成179立方以上高炉7座,建成万吨以上铸造企业25家。中高档铸件达到17.5万吨。煤、铁两大传统产业在技术装备、资源利用、环境保护等方面有了明显进步。非公有制经济得到长足发展,在全市经济总量中所占比重由34%提高到35.5%。旅游兴市战略顺利实施，建成专用公路14条、268公里,重点景区环境明显改善。全年接待游客219万人次。“2316”调产目标初见成效,65个项目完成投资25.5亿元，建成投产和部分投产35个。

　　3、城市规划建设管理取得新突破。完成了中心城市第三轮总体规划。“五大工程”成效显著,城市品位进一步提高，人居环境进一步改善。开通了8段街道,新增城市道路8.1公里；完成三个公园植树700多亩, 7段街道更新大树6827株,种植各类苗木49.6万株,草坪15.2万平方米,新增绿化面积1200亩。城市绿化覆盖率、绿地率和人均公共绿地面积分别达到35%、27%和8平方米，城市整体绿化水平达到省级园林城市标准；对市区主要街道80多座沿街建筑物进行了立面装饰和亮化；“两河”治理“2388”段初见成效,百丽园、西秀园已对市民开放。黄华街西区城中村改造顺利进行。城市管理力度加大。组建了城市管理行政执法局，开展了城市环境综合整治，城市管理迈入规范化、法制化轨道。

　　4、基础设施建设成效显著。全社会固定资产投资82.3亿元,增长20.8%。十大重点工程完成投资18.5亿元,占计划的100%。晋长高速路路基全线贯通。晋陵一级路陵川段建成通车。晋济高速路已经奠基开工。市综合馆、市体育场主体工程基本完成。污水处理厂开工建设。张峰水库完成控制性工程。“村村通”成效显著，建成通乡油路84公里,通村公路2403公里。

　　5、精神文明和民主法制建设继续加强。积极贯彻公民道德建设实施纲要，群众性文明创建活动取得成效，跨入了省级文明城市行列。各级政府自觉接受人大依法监督、政协民主监督，全年办理人大代表建议163件和政协委员提案240件。重视和加强人民群众来信来访，从源头上预防和治理腐败，政府系统廉政建设取得新成效。开展了“三打三防三创”活动，全市治安秩序良好，社会稳定。基层民主政治建设扎实推进。

　　6、城乡人民生活水平稳步提高。全市下岗职工基本生活费和离退休人员工资实现足额按时发放，城市低保全员覆盖，2.7万名低保对象全部得到最低生活保障。农村低保稳步推进。发放《再就业优惠证》1.11万个,“4050”再就业人数达938人，超计划91人。城镇失业登记完成省控指标。新解决农村温饱问题7000人，解决农村饮水困难6万人。

　　7、各项社会事业全面进步。组织实施国家、省、市级各类科技计划54项，开发新产品、新技术60个。积极推进义务教育，加快学校建设步伐。全市中小学危房改造基本完成，新建、改扩建农村寄宿制学校40余所。繁荣发展文化事业，一批优秀作品在全国、全省获奖。群众体育活动蓬勃开展，竞技体育水平稳步提高。人口、资源、环境工作得到加强。国防动员、双拥、民兵预备役和人防工作取得新的进展。广电、民政、文物、史志、气象、地震、档案、外事、侨务、民族、宗教、妇女、儿童、老龄、残疾人等各项社会事业都有新的发展。

　　8、抗击非典取得重大胜利。去年春天，面对突如其来的非典灾害，我们坚决贯彻落实全国和省“非典”防治工作的各项部署，沉着应对，果断处置。建立了强有力的组织指挥系统，构建了条块结合、盖边沉底的防控体系，统一调度人财物资源，保证了预防、诊治高效有序地进行。各级领导靠前指挥，亲自参战，广大人民万众一心，严防死守，迅速形成了县自为战、乡自为战、村自为战的防控局面，有效遏止了疫情的传播和扩散，夺取了抗非战役的重大胜利。这是具有光荣革命传统的晋城人民在新的发展时期，谱写的一曲“众志成城，敢打必胜”，应对重大突发事件的新篇章。同时，大力开展爱国卫生运动，积极推进农村新型合作医疗试点，加强公共卫生体系建设，使疫情防治工作制度化、规范化、法制化。

　　去年的发展使我们进一步深化了对经济社会发展规律的认识，更加深刻地体会到：一是必须毫不动摇地坚持以经济建设为中心，牢固树立发展意识和机遇意识。“发展是硬道理”，发展是解决一切问题的关键。“聚精会神搞建设，一心一意谋发展”，在任何时候、任何情况下都不能动摇。二是必须坚持以人为本，牢固树立和落实科学的发展观。发展不能就经济抓经济，而是要在集中力量抓好经济建设的同时，注重经济与社会、城市与农村、人与自然的统筹和谐，以实现全面、协调、可持续发展。三是必须充分尊重和发挥广大干部群众的首创精神和主观能动性。实践证明，只要充分尊重人民群众的首创精神，充分发挥人民群众的干劲、智慧和积极性，我们就没有克服不了的困难。四是必须坚持求真务实、真抓实干的作风。只有努力建设一支求真务实、真抓实干的干部队伍，崇尚发展，力戒空谈，件件工作抓落实，我们的事业才能大有希望。所有这些都是全市人民智慧的结晶，也必将成为晋城争先发展的宝贵财富和不竭动力。

　　各位代表,2003年我市在许多方面发生了令人可喜的变化，全市上下加快发展、争先发展的热情更高、信心更足、氛围更浓。虽然我们遇到的困难比预料的要多，但取得的成绩比预料的要好。成绩来之不易。这是省委、省政府和市委正确领导的结果，是市人大和市政协大力支持监督的结果，是全市人民团结奋斗、拼搏进取的结果。在此，我代表市人民政府向全市人民致以崇高的敬意！向各民主党派、人民团体、各界人士以及关心支持晋城发展的朋友们表示衷心的感谢！

　　在肯定成绩的同时，我们也清醒地看到，在前进道路上还存在不少困难和问题，政府工作还存在不少缺点和不足。主要表现在：主导产业单一的经济结构还没有发生根本性变化，低水平重复建设现象依然存在，结构调整任重道远；“三农”问题仍很突出，农民增收的难度很大；体制性障碍成为发展的最大“瓶颈”，国有、集体企业特别是资源型企业改革步履艰难；对外开放程度不高，民营经济步子不大，县域经济很不平衡；政府职能转变还不到位。与此同时，在发展中又暴露出一些新的矛盾，诸如电力紧张、运力不足、资金短缺、环境污染、社会就业等问题。对此，我们必须保持清醒的头脑，迎难而上，求真务实，去接受新的考验，迎接新的挑战。

　　二、2004年政府工作的总体要求和主要任务

　　各位代表，2004年是全面贯彻落实党的十六大和十六届三中全会精神的重要一年，是实现“十五”计划的关键一年，也是我市争先发展,建设经济强市、园林城市、一流文明城市和优秀旅游城市的全面攻坚之年。努力做好今年的政府工作，意义十分重大。

　　今年，市政府工作的总体要求是：以邓小平理论和“三个代表”重要思想为指导,全面贯彻党的十六大和十六届三中全会精神, 认真落实省委省政府的各项决策，围绕“一争两转五突破”的工作部署和“11445”的发展战略,紧紧扭住“四市”建设目标，突出争先发展、科学发展这个主题，坚持以人为本，牢固树立和落实科学的发展观和正确的政绩观，更加注重宏观调控，更加注重改革开放，更加注重“三农”问题，更加注重社会发展，更加注重统筹协调，组织和动员全市人民，抓住机遇，加快发展，为顺利实现我市新时期新阶段第一个目标，进而为全面建设小康社会奠定坚实的基础。

　　按照这一总体要求,今年全市经济社会发展的主要预期目标为：全市生产总值 223 亿元,增长11%，提前一年完成“十五”计划；财政总收入34亿元,增长14%;一般预算收入11.4亿元,增长11%;城镇居民人均可支配收入7800元,增长10%;农民人均纯收入3150 元,增长7%;城镇登记失业率控制在4.5%以内;人口自然增长率控制在7‰以内;市区空气质量二级以上天数保证达到160天,力争达到180天。

　　以上预期目标，是在认真分析当前宏观经济形势，充分考虑煤电油运等诸多因素的基础上，把速度与效益、需要与可能结合起来，加以综合平衡而确定的，是留有充分余地的。当然，在具体实施过程中，我们不能拘泥于甚至满足于这个预期目标，要瞻前顾后，审时度势，坚决落实中央的宏观调控政策，对经济运行实施“适时适度、有力有效”的调控，切实把广大人民群众加快发展的积极性引导好、保护好、发挥好，力争把工作做得更好一些，努力实现速度和结构、质量、效益相统一。

　　为此，我们必须全力抓好以下八个方面的工作：

　　1、大力推进结构调整，加速经济协调发展

　　牢固树立“工业立市”、“旅游兴市”理念，突出抓好传统产业的提升和新兴产业的培育，全力构筑晋城经济的“四梁八柱”，加快经济增长方式转变，推动经济运行质量提高。

　　全力推进四大基地建设。清洁煤基地要加快矿井建设,尽快使兰花三矿和大宁、王坡等一批骨干矿井按期投产。推广长壁和综采工艺,抓好筛分洗选,提高回采率和块炭率。电力基地在重点推进阳电二期2×60万千瓦项目、鲁能4×60万千瓦项目和50万伏输变电工程开工的同时,要集中力量加快兰花阳城、高都两个2×13.5万千瓦电厂的建设。全力推进春光4×30万千瓦热电联供一期工程和巴公4×5万千瓦项目开工。化工基地重点是抓好兰花阳城1830、天脊4060和晋丰3652项目,确保明年底形成200万吨尿素生产能力。铸造基地重点是推动冶铸联产共调,抓好开发区国际铸造园、泫氏、晨晖、群英、精工等中高档铸造扩建项目,力争中高档铸件产量突破50万吨。

　　大力发展接续产业。加大产品研发力度，积极发展精细化工、精密铸件和机加工业，用2-3年时间构筑晋城新兴工业框架体系。精细化工重点是抓好50万吨甲醇、20万吨乙醇以及三聚氰胺等项目。精密铸造重点是培育开发区铸造园、泫氏、晨晖等企业。机加工业重点是重组和培育太印、气门、群英、汉通、小笠原、金宝特等加工企业。同步加快建材、丝麻、饮品业的上档升级，培育新的经济增长点。

　　推进信息化建设。信息化是我市“四化”建设目标之一。要坚持以信息化带动工业化，以工业化促进信息化，加大实施信息化工程力度，在完善提高城域网的基础上，科学整合信息化资源，办好政府公务网和社会商务网，广泛应用信息技术，使之成为推动我市经济和社会发展的强大动力。

　　加快优秀旅游城市创建步伐。旅游业是我市最具发展潜力的朝阳产业，今年的省政府工作报告中已经明确提出，要发挥大同、晋城、运城“金三角”的区位优势，打造山西特色旅游业的新形象。我们必须紧紧抓住这一重要的历史机遇，大力实施“旅游兴市”战略，以旅游促开放、促发展。要坚持“政府主导，市场运作，重点突破，整体推进”的工作方针，加快旅游基础设施特别是主要景区通道建设，加快青莲寺、王莽岭和皇城相府三大景区开发建设，完善景区吃、住、行、游、购、娱等要素功能。理顺景区管理体制，加大旅游促销力度，整顿规范市场秩序, 培养造就优秀的旅游队伍，努力提高服务水平，全力打造“太行山水”旅游品牌。

　　发展现代服务业。制定并实施现代物流业发展规划,重点建设辐射能力强的专业特色市场,整顿和规范生产资料供应市场、农产品批发市场、小商品市场以及各类专业市场,积极发展连锁经营和物流配送,不断满足人民群众的消费需求,以扩大消费,拉动经济增长。加大对外贸出口企业的扶持力度,加快外贸出口商品基地建设,扩大各类企业的自营出口权,保持对外贸易的较快增长。积极发展房地产、法律、信息、咨询、家政等新兴服务业和各类中介组织。积极创新金融、保险体制,强化银企合作,为经济建设提供优质服务。

　　加快基础设施建设步伐。立足突破制约我市经济发展的瓶颈，经过认真筛选，确定了今年要集中力量抓好的16项重点工程。其中，续建8项，分别为晋长高速路、晋陵一级路、大宁煤矿、市综合馆、市体育场、市污水处理厂、两河治理和城中村改造；新建8项，分别是张峰水库、晋济高速路、端润一级路、市垃圾处理厂、阳电二期、晋城4×30万千瓦热电联供、晋城煤层气开发利用和市技校扩建工程。这些项目都要在确保质量和安全的前提下，精心组织，加快进度，按期竣工交付使用。同时认真做好鲁能4×60万千瓦、晋侯高速路、市委党校迁建、市图书馆和市档案馆5个项目的前期准备工作。

　　2、巩固提高农业基础地位，千方百计增加农民收入

　　农业是国民经济的基础。必须坚持统筹城乡发展和“多予、少取、放活”的方针，更多地关注农村，关心农业，关爱农民，采取切实有效措施，强化农业基础地位，努力增加农民收入。

　　扎实推进农业结构调整。突出抓好畜牧业的发展，扶持养殖大户,推广优良品种,加强畜禽防疫,实行科学养殖,在禽流感防治取得阶段性胜利的基础上,巩固扩大成果,确保畜牧业生产安全。经过今明两年的发展，猪、牛、羊饲养量要分别达到108万头、10万头和100万只，全力推动畜牧业规模化、产业化。同步推进蔬菜、果品、蚕茧、中药材、小杂粮基地建设。加快农业产业化步伐，力争培育一批具有一定规模和较强竞争力的农畜产品加工、储运、销售一体化龙头企业。推进农业标准化生产,建立完善农产品质量检测检验体系。大力发展农村中介组织和农民经纪人队伍,提高农民进入市场的组织化程度和抗御风险的能力。

　　加强农业基础设施建设。抓紧落实张峰水库开工建设。实施城市防洪、中小水库除险加固，加快东焦河、曹河、磨滩、秦家磨、湾则五个小水电项目建设。继续花大力气搞好农田水利基本建设，新增节水灌溉3万亩，治理水土流失15万亩，推广地膜覆盖、秸秆覆盖等技术，治旱兴农，提高粮食综合生产能力。

　　统筹城乡协调发展。加强小城镇规划建设，引导和鼓励农民向城镇集中。加强农村劳动力素质技能培训，认真落实农民工工资政策，决不能再发生新的拖欠。坚决执行新增文教卫生经费主要用于农村的政策规定，促进农村各项社会事业健康发展。

　　认真落实党在农村的各项政策。要万分重视粮食生产, 稳定粮食生产水平。要实行最严格的耕地保护制度, 稳定土地承包经营。继续深化农村税费改革,取消农业特产税,逐步降低农业税税率,今年要降低1个百分点,五年内取消农业税。推进粮食流通体制改革,落实对粮农的直补政策。健全农民负担监督机制，严肃查处损害农民利益的事件,切实维护好广大农民的根本利益。

　　今年，要重点为农民办好十件实事：一是投资4亿元，完成500个村1500公里“村村通”工程；二是由市政府出资500万元、六县（市、区）各出资300万元，建立农村教育发展专项资金；三是投资90万元，实施农民致富技能培训27万人次；四是巩固扩大泽州县农村新型合作医疗试点，做好阳城县试点准备工作；五是开展农民工劳务输出，培训就业1万人，向市外输出5000人；六是由市财政补助500万元，使500个行政村的群众能清晰地收看到央视和本省、本市的电视节目；七是投资1400万元，完成晋长、晋陵两条通道绿化和1700公里“村村通”道路绿化任务；八是市政府投入350万元，各县（市、区）投入546万元，对目前4.3万农村贫困人口全部实行低保；九是启动“1412”城镇化建设战略，逐步完成市区2.8万农民转户；十是新建90处饮水工程，解决6万人、9000头大牲畜饮水困难。

　　3、提升城市规划建设管理水平，塑造城市新形象

　　按照城市的目标定位，坚持高起点规划、高标准建设、高效能管理，进一步改善人居环境，提升城市品位。

　　强化城市规划的龙头作用。按照以人为本和经济与社会、人与自然协调发展的要求，搞好中心城市总体规划编制。同步进行县城和中心集镇统一规划，分步实施，尽快形成“1412”城镇发展格局，即1个中心城市、4个卫星城市、10个试点城镇和200个示范村。力争通过5—10年努力使全市城镇人口和城镇化水平由现在的86万人、39.3%，达到130万人、55%以上。

　　加快城市建设步伐。严格实施城市总体规划，不断加快城市建设步伐。重点实施“六大工程”。畅通工程：今年要打通4段、3公里城市道路，贯通36公里外环路，使城市道路更加畅通便捷。蓝天工程：推行清洁生产，集中治理锅炉污染，严格禁用散煤，规范使用型煤，综合整治市区主要污染源。加快热电联供和煤层气项目建设,扩大市区集中供热供气覆盖面，改善市区空气质量。碧水工程：按照“东河治污、西河变美”的总体目标，国庆节前完成凤台街至中原街2388米的“两河”治理一期工程，形成10万平米的水域面积和沿河景区。年底完成根治两河”的任务。启动东焦河水电工程，完成大坝60%的主体工程，实现部分蓄水，初步形成融青莲寺、珏山、丹河大桥、东焦河水库于一体的城市休闲渡假旅游景区。同步治理丹河,彻底关闭沿河“土小”企业，恢复丹河生态环境。绿化香化工程：根据创建园林城市的要求，加快18.3公里环城绿化建设，完成9.63公里城市新建道路绿化。开展全民义务种花种草和庭院绿化活动,建成10个绿化达标住宅小区，使城市绿地率、绿化覆盖率和人均公共绿地分别达到30%、38.5%和11平米。美化亮化工程：做好市区主要街道沿街建筑和“两河”沿线的美化亮化装饰工作。净化工程：以改善人居环境为目标,实施城市污水和垃圾无害化处理。建成污水处理厂,新建垃圾处理厂。同时要完成黄华街西区城中村改造工程,保证年内居民顺利回迁。坚持经营城市理念,按照“谁投资、谁受益”的原则,对经营性基础设施的所有权、经营权、广告权实行有偿使用或公开拍卖,逐步放开市政公用设施市场,实现投资主体多元化。

　　提高城市管理水平。重点是理顺城市管理体制，加快重心下移、事权下放，充分发挥社区的作用，推动城市管理取得新突破。抓好市区主干道环境综合整治，坚决拆除各类违章建筑。全面落实辖区管理和“门前三包”责任制，整治占道经营，规范户外广告，加强交通监管。特别是要解决好老城区小街小巷的硬化，垃圾清运和上厕难问题。强化城市管理宣传教育，逐步提高市民城市意识和城市化水平。

　　4、继续深化改革，进一步扩大对外开放

　　深化国有企业改革。认真贯彻十六届三中全会《决定》精神，按照现代企业制度的要求，大力推行国有企业产权制度改革。重点是积极推行参股、控股、破产、重组等方式，推动煤炭企业走集团化、规模化的路子。市里要抓好兰花、煤运、大宁等大型煤炭企业的联合，以资源为依托、资本为纽带、销售为龙头，积极组建晋城煤炭集团。各县（市、区）要抓县、乡骨干煤矿的联合,以县（市、区）为主筹建区域性煤炭集团。进而推动市、县联合，加快筹建无烟煤集团的步伐。今年市、县两级力争完成各自集团的筹建。要进一步搞好晋钢、太印、气门等市属企业改革重组,全面推进县属企业改革,大力发展国有、集体和非公有制资本相互参股的混合所有制经济。积极探索国有资产监管和经营的有效形式,确保国有资产保值增值。

　　推进乡村企业改革。以产权改革为核心，进一步加快乡村集体企业股份制改造。对资源型乡村企业，坚持依法管理矿产资源，依法规范采矿行为，积极推行资源有偿使用，努力做到资源有效利用、企业增加活力、环境得到保护、安全得到保障、农民得到实惠。

　　大力发展非公有制经济。坚定不移地落实加快发展非公有制经济“十八条”,全面放宽市场准入，切实让非公有制经济在投融资、税收、土地使用和对外贸易等方面享受同等国民待遇。对承担重点调产项目的非公有制企业，在资金、政策上给予大力支持。不断增强中小企业信用担保能力，为非公有制经济提供信贷支持，促进非公有制经济快速发展。

　　深化财政体制改革。建立完善公共财政体制,保障公共产品和服务的财政支撑。强化部门预算管理，推行政府网上采购。加强税收监管，严格依法征缴。调整财政资金投向，新增财力重点向农村倾斜，向交通、能源、水利、生态环境建设倾斜,实现财政向社会型的转变。

　　大力实施开放活市战略。牢固树立大晋城、大开放、大发展的新理念,从全局和战略的高度来认识并把握好历史机遇,积极开展招商引资活动。坚持“走出去”战略,充分发挥资源优势，强力吸引人才、技术、资金的流入，加快与山东、浙江等经济技术强省的合作步伐，积极融入中原经济圈，展开双边、多边和区域合作，搞好产业对接与经济互补。积极促进鲁能煤电化项目早日开工建设。坚持政府主导，突出企业主体，改进招商方式，落实激励政策，调动全社会招商引资的积极性，努力提高我市对外开放水平。

　　5、切实加强人口资源环境工作，促进可持续发展

　　加大环境保护力度。根据我市产业特点以及环保面临的压力和任务，今年要采取法律、经济、行政和技术手段相结合，实行“多管齐下”，动员全民参与，加大执法力度，大打一场改善环境质量攻坚战。市政府已列支2000万元，专项用于污染治理和实施“蓝天、碧水、生态”工程。首先把市区建成环境质量首善之区，进而带动和推进全市环境质量的改善。要继续加大“土小”企业关闭力度,特别对污染严重的焦化、冶炼行业，要按照省委、省政府的部署，实施专项清理整顿，强化城乡环境污染治理。对大气污染的两大污染物——可吸入颗粒物和二氧化硫进行综合治理，确保大气污染总量下降10%。加强丹河流域水污染防治，搞好沁河流域保护性开发，使全市水质污染程度好转12%。抓好重点生态工程建设,加快市区环城绿化和通道绿化，大力推进陵川、沁水生态示范区建设，努力使全市环保工作提高到一个新的水平。

　　加强资源的保护和利用。积极发展循环经济，大力开展节能降耗和综合利用。对煤炭等矿产资源要坚持依法保护，合理开发，总量控制，结构优化。对土地资源要实行更加严厉的保护措施，严格审批，加大复垦，确保耕地总量动态平衡。实施各种综合节水措施，不断提高水资源利用率，建设节水型社会。加大研发力度，综合利用“三废”，减少污染，节约资源。

　　做好人口与计划生育工作。加大人口和计划生育工作力度，建立和完善依法管理的工作机制，全面提高人口与计划生育的管理水平和服务水平。强化人口意识，严格控制人口过快增长，实行优生优育，不断提高出生人口素质。

　　6、统筹经济社会发展,促进各项社会事业全面进步

大力实施人才强市战略。必须树立人才是第一资源的观念，切实把人才强市战略纳入经济发展的大局，作为政府工作的重中之重，打破阻碍人才流动的体制性障碍，创新人才的工作机制，搭建高端人才创业平台。坚持培养与引进相结合，依托职业学院、技校和职中，大力培养我市经济建设急需的各类专业实用技术人才。今年要培训7万名农业技术骨干，启动农村小康建设人才集聚工程，用三年时间完成向全市2300多个村，每村选派一名大学生的任务。制定更加优惠的政策，把人才引进与项目、产业发展结合起来，创造各类人才“引得进、留得住、用得上”的机制，营造尊重知识、尊重人才，鼓励人们干事业、支持人们干成事业的良好氛围。

　　推进科技教育卫生事业。切实把教育放在优先发展的战略地位，巩固和扩大“两基”成果。市、县两级出资2300万元，建立农村教育发展资金，保证税费改革后，农村教育正常发展。完成100所高标准寄宿制小学的建设，保障偏远贫困地区农村孩子就学。开工建设中原街中学。积极发展职业教育，扩建职业中学和技工学校，进一步筹划高等教育。全面推进科技进步。各级政府和职能部门要加大对产品研发的领导，高度重视并支持研发工作，培养研发队伍。确立企业技术创新和科技投入的主体地位，不断强化与科研院所的合作，有条件的企业要积极建设自己的研发中心，围绕我市结构调整，突出发展精细化工、生物制药、机械加工、农产品加工等新兴产业，推动“四梁八柱”经济体系不断完善。加快农业科技推广体系建设，完善四级农技服务网络，把先进实用的农业科技送到千家万户，帮助广大农民增收致富。加强公共卫生设施建设，完善疾病防控和医疗救治体系。完成市急救中心、疾控中心和传染病医院的建设。加强农村卫生工作，稳步推进农村新型合作医疗制度试点，积极发展城市社区医疗服务，大力开展城乡爱国卫生运动。

　　加快发展文化产业。随着经济的发展和社会的全面进步，必须把文化事业和文化产业的发展摆到更加重要的位置。要大力发展社会主义先进文化，弘扬以爱国主义为核心的民族精神，高度重视并切实加强青少年思想道德教育。进一步发展城市文化和企业文化。繁荣文学艺术、新闻出版、广播影视业，实施精品战略，为广大群众提供更多的健康向上的文化产品。继续加强文化市场和互联网的建设与管理，持久开展“扫黄打非”，不断净化文化市场。要广泛开展全民健身运动，提高竞技体育水平，开好第三届市运会。积极发展档案、文物、史志、气象、测绘等事业,做好妇女、儿童、统计、档案、老龄等各项工作。

　　7、推进精神文明和民主法制建设，营造和谐稳定的社会环境

加强精神文明建设。认真实施公民道德建设纲要，引深精神文明创建活动。要按照“主题不变，内容不变，目标不变，时间不变”的要求，切实加强领导，巩固提高创建成果，为明年进入创建国家级文明城市先进市打下良好的基础。深入开展“建文明晋城市，做文明晋城人”活动，努力提高公民的思想道德和科学文化素质。

　　推进民主法制建设。各级政府要自觉接受同级人代会及其常委会的法律监督，接受人民政协的民主监督，听取民主党派、工商联、无党派人士和人民团体的意见，接受新闻舆论和社会公众监督，认真办理人大代表建议和政协委员提案。完善重大决策规则和程序，推进决策民主化、科学化。深入开展普法教育，搞好矛盾排查、司法调解，筑牢社会稳定的“第一道防线”。强化行政执法责任制和过错责任追究制，进一步完善政务公开、村务公开、厂务公开等基层民主管理制度，依法保障公民、法人和其它组织的合法权益。

　　全力维护社会稳定。加强公安政法队伍建设，强化社会治安综合治理，深入开展严打整治专项斗争。高度重视人民群众来信来访工作，及时化解各种矛盾。做好国家安全和民族宗教工作。加强国防教育，开展“双拥”工作和“双服务”活动，搞好民兵预备役和人民防空建设，切实提高国防动员能力.

　　8、加大就业和社会保障力度，努力改善人民生活

　　切实做好就业再就业工作。按照中央和省关于促进就业和再就业的要求,严格各级政府扩大就业的责任，实施积极的就业政策,推行灵活的就业方式。完善就业服务体系，搞好就业技能培训、职业介绍和就业指导，加强对就业再就业的督查落实。大力发展劳动密集型产业、非公有制经济和第三产业,拓展就业渠道。全市城镇新增就业岗位3万个，安置“4050”人员830人。

　　完善社会保障体系。认真落实“两个确保”，加强 “三条保障线”的衔接。完善职工基本养老保险，稳步推进国有企业下岗职工基本生活保障向失业保险并轨。依法扩大社保覆盖面，加强对各类参保企业保险费的征缴。足额落实城乡低保配套资金，完善城乡低保制度。

　　努力提高城乡居民收入。改革分配制度，规范分配秩序。各类企业要严格执行最低工资制度，严禁违法拖欠和克扣工人工资，健全按时支付农民工工资机制。保证机关事业单位特别是教师工资按时足额发放。高度重视和关爱弱势群体，广泛开展扶贫济困送温暖活动，关心支持残疾人事业，让他们真正感受到党和政府的关怀。

　　积极推进社区建设。按照“简政放权，重心下移，权责统一，属地管理”的原则，切实搞好社区建设，增强社区服务功能。建设面向广大居民、面向特殊群体、面向下岗职工的社会化服务平台。提高社区自我管理能力，努力建设管理有序、文明祥和的新型社区。

　　切实抓好安全生产。安全生产重于泰山。必须强化安全生产责任制，认真落实各项安全措施，坚决遏制重特大事故发生。坚持抓领导、领导抓，明确企业的安全生产责任主体地位；坚持抓培训，抓管理，全面推进煤矿安全质量标准化工作，年内完成煤矿双回路和瓦斯数字监测两大基础设施建设；坚持抓落实，抓追究，严肃查处各类责任事故。强化安全专项整治，对煤矿、交通、易燃易爆、森林防火、防汛等高危行业，要全面落实安全措施，加强安全监督，确保安全生产。

　　各位代表，2004年市政府将为城市居民办好15件实事：一是综合整治市区污染源，保证市区空气质量二级以上天数160天，力争180天；二是完成集中供热三期工程，新增供热面积80万平米；三是新增街头绿地12000平米，建成黄华街“鲜花一条街”；四是进行环城绿化，使城市绿化覆盖率达38.5%；五是拓宽690米前进路，缓解老城交通压力；六是完成泽州路南段1100米，连接晋长高速；七是建设黄华街南段600米，连通回迁安置小区；八是开通东环路中段739米，完善市区交通环网；九是完成凤台街、泽州路、黄华街11000米街道和29座建筑的美化亮化；十是新建放心夜市20个、蔬菜早市15个；十一是新建一中艺术体育馆、教学楼、塑胶跑道，达到标准化示范高中的水平；十二是完成五中教学楼、中原街小学和黄华街西区城中村改造新建小学；十三是市区内建设高标准公厕20个、垃圾收集站17个，硬化老城小街小巷6640平米；十四是完成“两河”和城中村回迁安置小区30万平米；十五是新建市传染病医院和市疾控中心。

　　面对新的形势和任务，必须加强政府自身建设，尽快形成行为规范、运转协调、公正透明、廉洁高效的行政管理体制和运行机制，树立人民政府新形象。

　　1、坚持创优环境，建设诚信晋城。深入开展以“优化发展环境,建设诚信晋城”为主题的创优活动,大力推行政务公开,实施阳光作业,减少审批事项和审批程序，提高行政效率，建设诚信政府、服务政府、法制政府。继续整顿规范市场秩序，严厉打击制售假冒伪劣行为。不断加强工商和质量技术监督,促进企业诚信经营。完善项目招投标、政府采购等制度,制止不正当竞争。加快全社会信用体系建设,完善激励守信、惩戒失信的社会信用监督机制,努力打造“诚信晋城”。

　　2、坚持依法行政，加快职能转变。以贯彻实施《行政许可法》为契机，加强对公务员的培训、教育、管理和监督，认真落实行政执法责任制和过错责任追究制，保证政令畅通，令行禁止。突出人本理念和发展理念，完善政府经济调节、市场监管、社会管理和公共服务的职能。按照省委省政府的统一部署，如期完成新一轮机构改革任务，更好地服务经济建设、服务投资主体、服务人民群众。全力推进依法行政，认真执行行政复议、行政诉讼、行政赔偿、行政许可等一系列法律法规，加快建立权责明确、行为规范、监督有效、保障有力的执法体制，努力做到有权必有责、用权受监督、侵权要赔偿。

　　3、坚持执政为民，切实改进作风。强化宗旨观念，切实把一切工作的出发点和归宿,放在维护全市218万人民的根本利益上。对关系群众根本利益的事要带着感情，办实办好。对群众关注的热点难点问题要亲力亲为，妥善解决，真正做到权为民所用、情为民所系、利为民所谋。大力弘扬求真务实的作风，求晋城发展之真，务兴市富民之实，坚决克服官僚主义、形式主义。深入开展行风评议，突出抓整改、抓落实，力争把各项工作做得更好。坚持正确的政绩观，保持只争朝夕的紧迫感，扎扎实实做好本职工作，以良好的实绩取信于民，让人民群众从发展的成果中得到实惠。

　　4、坚持艰苦奋斗，做到廉洁勤政。牢记“两个务必”，坚持不懈地进行党风廉政建设和反腐败教育，增强拒腐防变意识。认真落实“四大纪律、八项注意”和廉洁自律的各项规定，自觉遵守《党内监督条例》和《纪律处分条例》，规范从政行为，做到廉洁勤政。坚持标本兼治，强化行政监察和审计监督，从源头上预防和治理腐败。坚决查处违法违纪案件，保持公务员队伍的纯洁性。认真落实党风廉政建设责任制，以清新、廉洁的政风，带动整个社会风气的根本好转。

　　2004年是“四市”建设攻坚克难之年。今年的政府工作就是要瞄准“四市”建设目标，突出争先发展这个主题，扭住结构调整这条主线，做好八项工作，办好惠及城乡人民的二十五件实事，努力推动经济社会全面、协调、可持续发展。

　　各位代表，当前我市已经进入加快发展的关键时期，时间紧迫，任务艰巨，责任重大。让我们紧密团结在以胡锦涛同志为总书记的党中央周围，高举邓小平理论伟大旗帜，全面实践“三个代表”重要思想，在中共晋城市委的领导下，以更加科学的态度、更加扎实的作风，适应新形势，迎接新挑战，再攀新高峰，为早日实现全面建设小康社会的宏伟目标而努力奋斗！

　　让我们共同携起手来，建设更加美丽的城市，创造更加美好的生活！
